# Coronary Button Pseudoaneurysm Resolution After Aortic Root Replacement Upon Cessation of Anticoagulation

**DOI:** 10.1016/j.jaccas.2025.106670

**Published:** 2026-01-28

**Authors:** Lauren Bougioukas, Omar A. Jarral, William Y. Shi

**Affiliations:** Department of Cardiovascular and Thoracic Surgery, Northwell Health, New York, New York, USA

**Keywords:** anticoagulation, aorta, imaging

## Abstract

**Background:**

Anticoagulation after aortic surgery is associated with an elevated risk of pseudoaneurysm formation.

**Case Summary:**

We present an unusual case of a patient who, after a Bentall procedure, developed a right coronary button pseudoaneurysm after commencement of anticoagulation. After cessation of anticoagulation, serial computed tomography demonstrated complete resolution of this pseudoaneurysm, negating the need for reoperation.

**Discussion:**

This case adds to the scant literature describing spontaneous resolution of post-Bentall pseudoaneurysms, particularly those involving the coronary buttons. To date, most reports emphasize percutaneous or surgical repair, and as such, data on benign courses under conservative management are sparse.

**Take-Home Messages:**

Anticoagulation may initiate and propagate early coronary button pseudoaneurysms after aortic root surgery. Not all pseudoaneurysms after aortic root surgery need to be treated with procedural intervention. Nonoperative management with computed tomography surveillance, cessation of anticoagulation, and strict blood pressure management should be considered for treatment of pseudoaneurysms.

## Graphical Abstract

**Figure:**
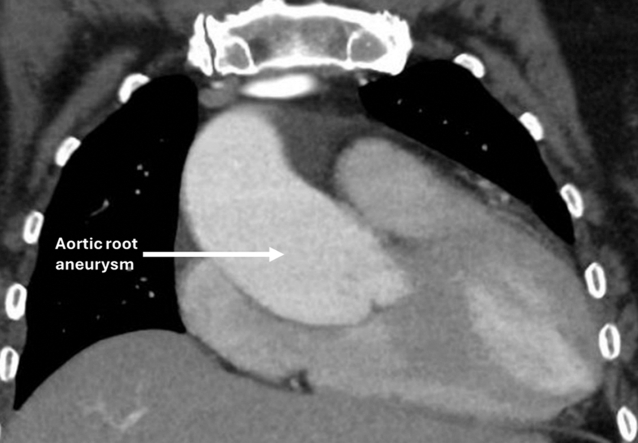

## History of Presentation

A 65-year-old female presented to our center with shortness of breath. Her past medical history was significant for hypertension, hyperlipidemia, and being an active smoker.

**Figure 1:**
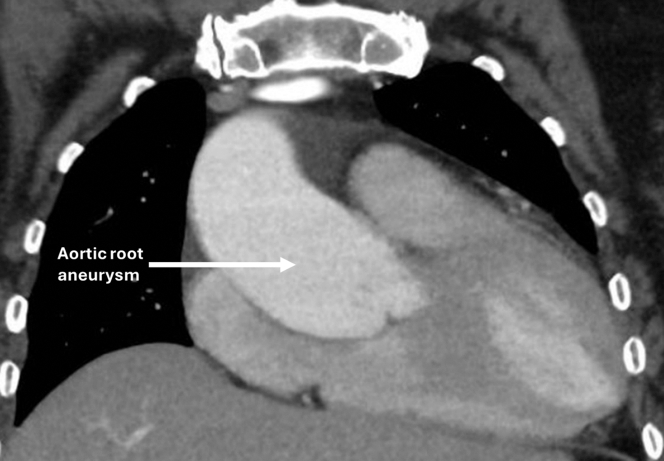
Computed Tomography of the Chest Demonstrating a Dilated Aortic Root of 5.3 cm

**Figure 2:**
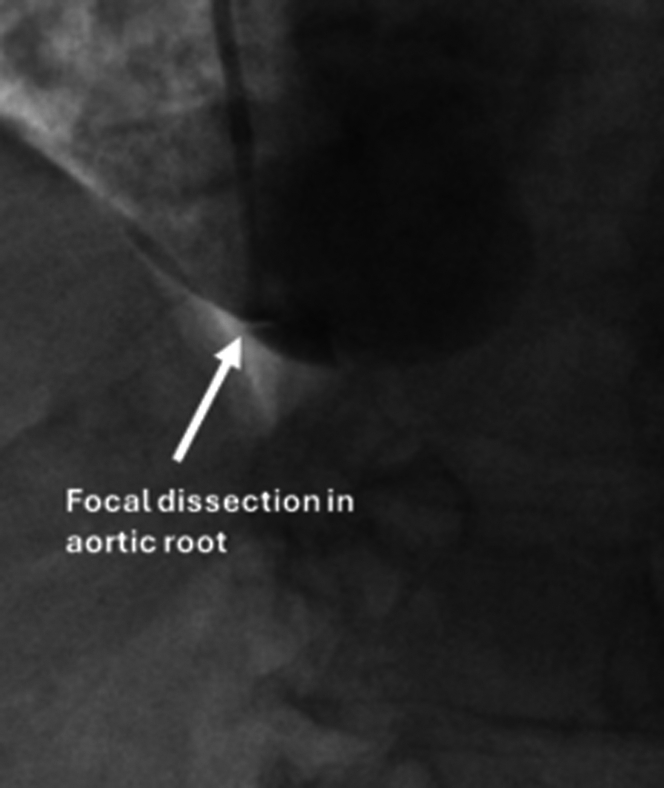
Iatrogenic Focal Aortic Dissection Occurring During Coronary Angiography

Transthoracic echocardiography demonstrated severe aortic regurgitation. Computed tomography (CT) of the chest demonstrated a dilated aortic root of 5.3 cm (Figure 1). She underwent preoperative coronary angiography which demonstrated nonobstructive coronary artery disease; however, the angiogram resulted in an iatrogenic focal dissection at the aortic root (Figure 2).

The patient had refractory chest pain and was transported emergently to the operating room where a transesophageal echocardiogram demonstrated that the dissection had propagated to become a Stanford Type A dissection extending into the aortic arch.

The patient thereafter underwent an aortic root replacement (Modified Bentall operation)—utilizing a 25-mm Konect tissue valved conduit—and an aortic hemiarch replacement under deep hypothermic circulatory arrest.

**Figure 3:**
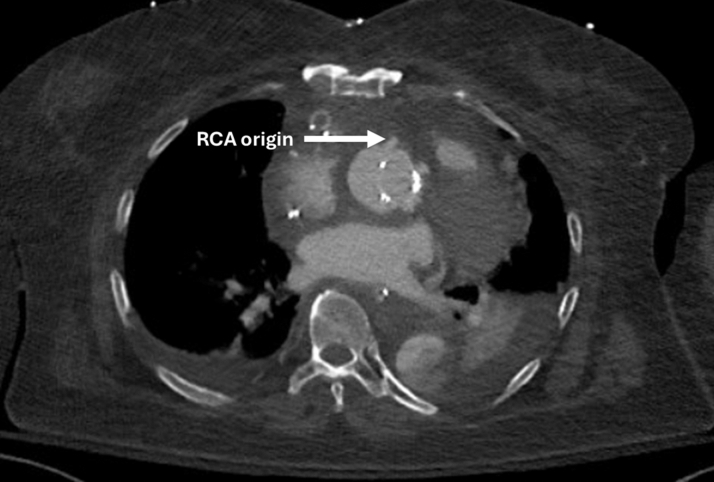
Postoperative CT Scan 1 Week After Surgery Demonstrating a Satisfactory Repair With No Concerning Features The origin of the reimplanted right coronary artery (RCA) is demonstrated.

Postoperatively, her initial course was uncomplicated, being extubated on postoperative day 2 and transferred to the step-down unit on day 3. However, her hospitalization was prolonged due to poor mobility and physical deconditioning. At 1 week postoperatively, she underwent a routine postoperative CT angiogram which demonstrated a successful repair with no concerning features (Figure 3).

On day 10, she was discharged to a rehabilitation center. Here, she was noted to have arm swelling, and doppler ultrasound examination of the upper limb demonstrated nonocclusive thrombus of the left subclavian vein. At this time, she also exhibited paroxysms of atrial fibrillation. As such, at 2 weeks postoperatively, she was commenced on apixaban at 5 mg twice a day by the rehabilitation clinical team.

## Investigations

**Figure 4:**
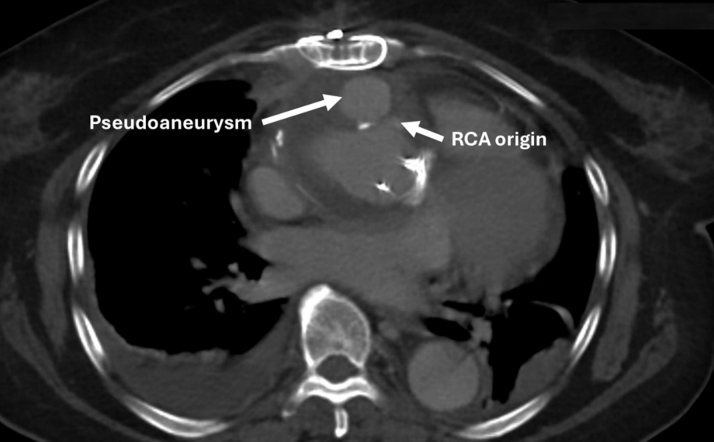
Appearance of an Aortic Pseudoaneurysm Anteriorly, Adjacent to the Site of the Right Coronary Artery Button Reimplantation

At 4 weeks postoperatively, she experienced some intermittent shortness of breath, prompting a repeat CT chest which demonstrated a pseudoaneurysm (PSA) anterior to the neo-ascending aorta (Figure 4).

## Management

Her anticoagulation was thereafter immediately ceased, and she remained solely on aspirin 81 mg daily. Beta blocker therapy was uptitrated, and strict blood pressure management was instituted so as to ensure her systolic blood pressure was maintained below 120 mmHg.

Given that she remained relatively debilitated at that time, it was felt she would present a poor candidate for early reoperative surgery to repair the PSA. Our institution's Aortic Team deemed surveillance to be a reasonable alternative.

## Outcome and Follow-Up

**Figure 5:**
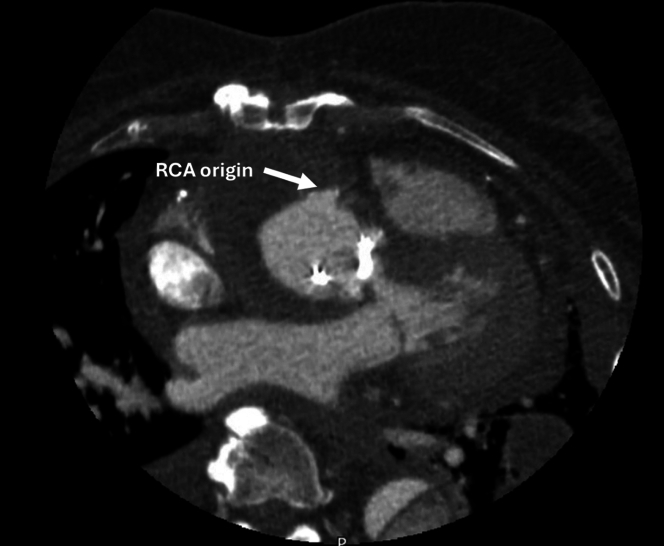
Repeat Cardiac Gated Computed Tomography 4 Months Later Demonstrating Resolution of the Pseudoaneurysm

Repeat CT aortogram was performed at 4 months after the initial surgery (3 months after cessation of anticoagulation and institution of strict blood pressure management). Here, the PSA had resolved (Figure 5). The patient thereafter continued to make a satisfactory functional recovery with plans for ongoing CT surveillance.

## Discussion

This case underscores a vexing but common postoperative scenario: the tension between the need for anticoagulation after a complex aortic root surgery and the risk of bleeding-related complications at fresh suture lines.

The available literature linking systemic anticoagulation to the development or persistence of postoperative PSAs is limited to case reports, small series, and expert opinion rather than prospective or controlled data, but these sources consistently flag anticoagulation as a biologically plausible risk factor that can exacerbate early suture-line bleeding, hematoma formation, and failure of micro-hemostasis at vulnerable anastomotic sites leading to PSA formation.^1^^,^^2^

Coronary button anastomoses, in particular, represent structurally vulnerable sites where tissue fragility, suture-line tension, and pressurized flow converge. The development of a PSA at this location after the initiation of systemic anticoagulation raises the possibility that pharmacologic anticoagulation may exacerbate micro-leaks at the coronary button, preventing effective hemostatic sealing in the early phase of graft incorporation.

Various methods to prevent PSA formation include creating large coronary buttons if the buttons are calcified; mobilizing buttons fully to decrease tension; preventing pseudoknot formation; obtaining full-thickness bites; folding graft material at the anastomosis to decrease tension on the suture line; reinforcing the anastomosis with a second layer of suture, Teflon, pledgets, or pericardium; and testing the anastomosis under pressure. If there is concern intraoperatively about PSA formation, transesophageal echocardiography can provide real-time imaging, although it is operator-dependent and has limited windows. Postoperatively, in high-risk cases (ie, cases with higher-than-average bleeding risk), electrocardiography-gated CT angiography is a high-resolution scan that can be obtained before discharge. Alternatively, magnetic resonance angiography is a longer scan with lower resolution but may be better suited for patients with renal dysfunction or those who cannot tolerate contrast.

The complete radiological resolution of the PSA after cessation of anticoagulation and institution of rigorous blood pressure control challenges the prevailing dogma that all postoperative aortic PSAs mandate urgent reoperation.^3^, ^4^, ^5^ While surgery remains the gold standard—particularly in symptomatic patients or those with expanding lesions—this case illustrates that a carefully selected, high-risk cohort may benefit from temporizing or even definitive nonoperative management. This highlights the importance of individualized decision-making in multidisciplinary “aortic team” settings, where operative risk, frailty, and competing morbidities must be weighed against the natural history of the lesion.

This case adds to the scant literature describing spontaneous resolution of post-Bentall PSAs, particularly those involving the coronary buttons. To date, most reports emphasize catastrophic rupture or describe percutaneous or surgical repair, and as such, data on benign courses under conservative management are sparse.^4^, ^5^, ^6^, ^7^, ^8^, ^9^, ^10^ The observation of resolution in this case suggests that not all PSAs follow an inexorably progressive trajectory. Rather, their biology may be heterogeneous, influenced by local tissue quality, hemodynamics, and systemic factors such as anticoagulation and hemodynamic status.

This case raises a testable hypothesis regarding the pathophysiologic role of anticoagulation in the genesis and persistence of early PSAs. Prospective data are lacking, but registry analyses could explore whether anticoagulated patients experience a higher incidence of button-related PSAs after root replacement. Our case also invites reconsideration of surveillance strategies. With high-resolution CT imaging now widely available, interval imaging may allow safe monitoring in select patients, sparing them the risk of complex redo root or coronary reimplantation surgery. Such an approach, if validated, could refine postoperative surveillance algorithms and patient counseling.

## Conclusions

Ultimately, this report contributes to the broader dialog about balancing surgical dogma with individualized, evidence-informed care. For the surgeons, it reinforces the importance of vigilance in postoperative surveillance, thoughtful and conservative integration of anticoagulation into recovery pathways, and willingness to consider nonoperative strategies in patients for whom reoperation carries prohibitive risk.

## Funding Support and Author Disclosures

The authors have reported that they have no relationships relevant to the contents of this paper to disclose.

**Take-Home Messages**

• Anticoagulation may initiate and propagate early coronary button pseudoaneurysms after aortic root surgery.
• Not all pseudoaneurysms after aortic root surgery need to be treated with procedural intervention.
• Nonoperative management with computed tomography surveillance, cessation of anticoagulation, and strict blood pressure management should be considered for treatment of pseudoaneurysms.
